# Expression of immune regulatory genes correlate with the abundance of specific Clostridiales and Verrucomicrobia species in the equine ileum and cecum

**DOI:** 10.1038/s41598-019-49081-5

**Published:** 2019-09-03

**Authors:** F. Lindenberg, L. Krych, J. Fielden, W. Kot, H. Frøkiær, G. van Galen, D. S. Nielsen, A. K. Hansen

**Affiliations:** 1 Brogaarden Aps, Lynge, Denmark; 2 University of Copenhagen, Faculty of Health and Medical Sciences, Department of Veterinary and Animal Sciences, Copenhagen, Denmark; 3 University of Copenhagen, Faculty of Health and Medical Sciences, Department of Veterinary Clinical Sciences, Copenhagen, Denmark; 4 University of Copenhagen, Faculty of Sciences, Department of Food Science, Copenhagen, Denmark; 5 Department of Environmental Sciences, Aarhus University, Aarhus, Denmark

**Keywords:** Mucosal immunology, Microbiome

## Abstract

Billions of bacteria inhabit the gastrointestinal tract. Immune-microbial cross talk is responsible for immunological homeostasis, and symbiotic microbial species induce regulatory immunity, which helps to control the inflammation levels. In this study we aimed to identify species within the equine intestinal microbiota with the potential to induce regulatory immunity. These could be future targets for preventing or treating low-grade chronic inflammation occurring as a result of intestinal microbial changes and disruption of the homeostasis. 16S rRNA gene amplicon sequencing was performed on samples of intestinal microbial content from ileum, cecum, and colon of 24 healthy horses obtained from an abattoir. Expression of genes coding for IL-6, IL-10, IL-12, IL-17, 18 s, TNFα, TGFβ, and Foxp3 in the ileum and mesenteric lymph nodes was measured by qPCR. Intestinal microbiota composition was significantly different in the cecum and colon compared to the ileum, which contains large abundances of Proteobacteria. Especially members of the Clostridiales order correlated positively with the regulatory T-cell transcription factor Foxp3 and so did the phylum Verrucomicrobia. We conclude that Clostridiales and Verrucomicrobia have the potential to induce regulatory immunity and are possible targets for intestinal microbial interventions aiming at regulatory immunity improvement.

## Introduction

As in other mammals, the equine intestine is inhabited by billions of bacteria. Host-microbial interactions and pro- and anti-inflammatory responses elicited in response to intestinal bacteria are crucial in establishing and maintaining intestinal microbiota homeostasis^1–4^. In mammals, this homeostasis is mediated through interactions between microbial-associated molecular patterns (MAMPs), *e*.*g*., bacterial lipopolysaccharides (LPS) and lipoproteins, and pattern recognition receptors (PRRs) on the epithelial and immune cells in the gastrointestinal tract (GIT). Through these interactions differentiation of regulatory T-cells (Treg) mediate oral tolerance towards commensal microbes forming the intestinal microbiota^5^. Dietary changes and other environmental perturbations may cause shifts in the microbial balance, as shown in humans, mice, and horses^6–8^. Changes in microbial composition, such as the Firmicutes:Bacteroides ratio, termed dysbiosis, and disruption of the immunological homeostasis has been associated with inflammatory diseases both within the GIT and systemically in humans, animal models and livestock^9–16^. Dysbiosis has also been suggested as a predictor of post weaning diarrhea in pigs^17^. Consequently, shifting the microbiota towards a composition that stimulates a more immunosuppressive response dominated by Tregs and anti-inflammatory cytokines, such as interleukin 10 (IL-10), transforming growth factor beta (TGF-β) and transcription factor Forkhead box P3 (FoxP3) has been linked to prevention of disease development and amelioration of symptoms of inflammatory diseases in humans and animals^18–21^. The intestinal microbiota may, therefore, be regarded as a potential target to treat and prevent conditions governed by low-grade chronic inflammation in and outside of the GIT.

Microbiota disturbances leading to disease are also known in equine medicine. Starch overload of the cecum and colon with subsequent pH drop leading to the death of vast microbial populations, gastrointestinal tympani, diarrhea, and endotoxemia has been associated with intestinal microbiota dysbiosis^22,23^. Also, colitis and colonic obstruction have been linked to changes in the microbial composition^24,25^. Development of laminitis as a result of starch overload^23,26^, suggests that changes in intestinal microbiota may also influence diseases outside of the equine GIT. Many horses suffer from equine metabolic syndrome (EMS), which shares several similarities with the human disease of the same name, most importantly chronic low-grade inflammation^27–29^. A contributing factor to the chronic low-grade inflammation in human metabolic syndrome is microbiota dysbiosis, *i*.*e*., smaller changes in intestinal microbiota composition leading to altered immune-microbial homeostasis and increased inflammatory state^9,10,14^.

The availability of new sequencing technologies, which allow for the entire microbiome to be studied including the strict anaerobes, have increased the understanding of the equine intestinal microbiota importance. However, available studies seeking to establish a core microbiota in horses have unfortunately been inconsistent in profiling methods, sampling regions, and inclusion criteria^30–39^. Strong correlations between specific intestinal bacteria and key inflammatory parameters are well documented in other species, *i*.*e*., mice^20,40,41^, rats^42^, and humans^43–45^. An example of this is the induction of Treg differentiation and reduced inflammatory levels through up-regulation of intestinal bacteria with a well-known anti-inflammatory effect such as *Lactobacillus* spp., *Bifidobacterium* spp., and various Clostridiales spp. through the addition of oligosaccharides to the diet^46–48^. In the intestinal microbiota of overweight individuals, the ration of Firmicutes to Bacteroides is skewed with a reduction in Bacteroidetes and an increase in Firmicutes^6,49^. It is currently unknown whether similar effects of bacteria on systemic inflammatory status can be obtained in horses. In order to investigate this, basal information is needed on which members of the equine intestinal microbiota exert either a pro- or an anti-inflammatory impact on the immune system.

The objective of the present study was therefore to identify specific bacteria or clusters of bacteria in the equine GIT, which are related to a regulatory response and therefore may be potential targets for improvement of the regulatory immunity.

## Method

### Collection of samples

Samples were collected post mortem from healthy horses at an abbatoir (Knorrevängen Slakt in Glimåkra Sweden). Only horses that were considered healthy by the veterinarian were included in the study. In total samples were collected from 24 horses between June 30^th^ and July 28^th,^ 2014. The horses were of mixed breeds, 17 mare and 6 geldings and aged between 2 and 21 (mean +/−SD: 11.09 +/−5.03). Samples of intestinal content for microbial analysis were collected from the ileum (approximately 15 cm oral to the ileocecal valve), the cecum and the colon (left ventral colon just oral from the pelvic flexure). Samples were stored in a mobile freezing box at −20 °C until arrival at the laboratory approximately two hours later, where they were immediately frozen at −80 °C. Samples of the ileal mucosa were taken to investigate the local immune reaction in the ileum wall, which is very dense in immune tissue^50^. Also samples from the lymph nodes (MLN) at the root of the mesentery were taken to investigate the general immune reaction in the intestine. Samples were stored separately in RNAlater (ThermoFischer Scientific, Denmark) for gene expression analysis. Samples were stored at 5 °C for 24 hours, after which the excess RNAlater was removed, and the samples were stored at −80 °C.

### Analysis of microbial composition

#### Extraction of DNA

Before extracting Deoxyribonucleic acid (DNA) from the intestinal content the samples were thawed, and 20 grams were suspended in 40 ml sterile milli-Q water in a stomacher bag with filter and processed in a stomacher (Seward 80 BA 7020 Stomacher Lab Blender, Seward, West Sussex, United Kingdom) in 2 min at maximum speed. A volume of 200 μl was then used for DNA-extraction using the MoBio Power Soil Kit (MoBio QIAGEN Nordic, Copenhagen) following the instructions of the manufacturer.

#### Sequencing of the intestinal microbiota

The concentration and purity of extracted DNA in each sample was measured using Nanodrop 2000 (ThermoFischer Scientific, Denmark), and concentration was normalised to ~20 ng/μl. The V3-V4 region (~460 bp) of the 16S ribosomal ribonucleic acid (rRNA) gene was amplified using primers compatible with Nextera Index Kit (Illumina) (nxt341_F: 5′-TCGTCGGCAG CGTCAGATGT GTATAAGAGA CAGCCTAYGG GRBGCASCAG-3′and nxt806_R: 5′-GTCTCGTGGG CTCGGAGATG TGTATAAGAG ACAGGGACTA CNNGGGTATCTAAT-3′). Polymerase chain reaction (PCR) reactions containing 12 μl AccuPrimeTM SuperMix II (Life Technologies, CA, USA), 0.5 μl of each primer (10 μM), 5 μl of genomic DNA (~20 ng/μl), and nuclease-free water to a total volume of 20 µl were run on a SureCycler 8800 (Agilent, CA, USA). Cycling conditions applied were: Denaturation at 95 °C for 2 min; 33 cycles of 95 °C for 15 sec, 55 °C for 15 sec and 68 °C for 40 sec; followed by final elongation at 68 °C for 5 min. To incorporate primers with adapters and indexes PCR reactions contained 12 μl Phusion High-Fidelity PCR Master Mix (Thermo Fisher Scientific, USA, MA), 2 μl corresponding P5 and P7 primer (Nextera Index Kit), 2 μl PCR product and nuclease-free water for a total volume of 25 μl. Cycling conditions applied were: 98 °C for 1 min; 13 cycles of 98 °C for 10 sec, 55 °C for 20 sec and 72 °C for 20 sec; elongation at 72 °C for 5 min. The amplified fragments with adapters and tags were purified using AMPure XP beads (Beckman Coulter Genomic, CA, USA). Prior to library pooling clean constructs were quantified using a Qubit fluorometer (Invitrogen, Carlsbad, CA, USA) and mixed in approximately equal concentrations to ensure even representation of reads per sample followed by 250 bp pair-ended MiSeq (Illumina) sequencing performed according to the instructions of the manufacturer.

#### Sequencing data analysis

The raw dataset containing pair-ended reads with corresponding quality scores were joined, trimmed, filtered from chimeric reads and clustered to operational taxonomic units (OTUs) as previously described^51^. The Greengenes (13.8) rRNA gene collection was used as a reference database. Quantitative Insight Into Microbial Ecology (QIIME) open source software packages (1.7.0, 1.8.0, and 1.9.1(Various versions of QIIME dealt with different bugs making some of the tests more accurate in older versions of QIIME or more applicable for given format of the file.))^52,53^ were used for the analysis as previously described in details^51^ with the following adjustment: the subsampling was performed using 10,000 reads per sample.

### Gene expression analysis

Ribonucleic acid (RNA) was extracted from both the MLNs and the ileal mucosa samples using the MagMAX™-96 Total RNA Isolation Kit (Thermo Fischer Scientific). Briefly tissue samples were thawed on ice and transferred to FastPrep® tubes containing 600–700 μl lysis buffer (MagMAX™-96 Total RNA Isolation Kit), 5 μl mercaptoethanol and 0.6 mg acid-washed glass beads (<106 μm, Sigma Life Science, Missouri, USA). The samples were homogenized using a Fast-Prep machine (Fast Prep®-24, MP Biomedicals, CA, USA) at speed 6.5 m/s for 45 sec for six runs. Subsequently, RNA was extracted from the supernatant on the MagMAX™ Express magnetic particle processor according to the manufacturer’s manual. After purification, the RNA concentration was determined by spectrophotometry at 260 nm using a NanoDrop 2000 (Thermo Fischer Scientific, Denmark) and RNA concentration was normalised to ~500 ng/μl. Complementary deoxyribonucleic acid (cDNA) synthesis was performed using the High-Capacity cDNA Reverse Transcriptase kit and GeneAmp PCR 9700 system (Applied Biosystems, Thermo Fischer Scientific, Denmark). Specialized equine probes were obtained from Applied Biosystems, Thermo Fischer Scientific for the genes Interleukin-6 (*il6)*, Interleukin-10 *(il10)*, Interleukin-12 *(il12)*, Interleukin-17 *(il17)*, Tranforming Growth Factor *(tgfb)*, Tumor Necrosis Factor-alpha *(tnfa)*, Forkhead Box P3 *(foxp3)* and *18* *s* (probe IDs: Ec03468680_m1, Ec03468647_m1, Ec03468747_m1, Ec03470096_m1, Ec03468030_m1, Ec03467871_m1, Ec04319948_m1, 4333760F). Quantitative PCR (qPCR) was performed on both tissues (MLN and mucosa) according to the manufacturer’s manuals using the TaqMan Gene expression Assay (Thermo Fischer Scientific, Denmark) and the 18S rRNA gene was used as reference. The amplification data was analysed using the StepOne v2.3 software (Applied Biosystems, Fischer Scientific Denmark) to obtain threshold cycle (C_T_) values. Quality check of the amplification data was performed and samples flagged NOSIGNAL (No detectable level of fluorescence), NOAMP (No amplification), BLFAIL (Baseline can not be fitted for the well) were excluded from the analysis.

### Statistical analysis

The differences in taxa abundance between compartments were estimated with a statistic framework: analysis of composition of microbes (ANCOM)^54^. The rest of the statistical analysis was performed using SAS University Edition (SAS Institute Inc. NC, USA) and GraphPad (GraphPad Software, Inc. La Jolla, CA, USA).

A one-way analysis of variance (ANOVA) using normalized OTU-tables (10,000 reads per sample) was used to analyse the differences in abundance of all phyla comprising 0.1% or more of the total recovered sequences in at least one compartment between the three compartments. From the qPCR analysis, threshold cycle (Ct) values were obtained and normalized to the reference genes. The relative expression of the anti-inflammatory genes *il10*, *tgfb*, and *foxp3*, the pro-inflammatory genes *il12*, *il17*, *tnfa*, and *il6*, were correlated (Spearman Rank Correlation) with the bacterial abundances to assess a possible relationship between intestinal microbiota and T-cell polarization. An analysis was done both on Phylum and genus level. Abundances in ileum were correlated to gene expression in both the mucosa and MLN and abundances in cecum and colon were correlated to gene expression in the MLN. Only genera present in at least half of the samples and with abundances above 0.1% of total recovered sequences in the respective compartment (ileum, cecum, colon) were included in the analysis. For each cytokine, 46, 41, and 40 correlations were performed in the ileum, cecum, and colon, respectively, and FDR values were calculated.

## Results

### Expression of specific regulatory genes in the ileum and MLN correlates with abundances of Verrucomicrobia and specific Clostridiales spp

With few exceptions, gene-expression of all genes was measured in all animals (Supplementary Fig. S1). The initial correlation analysis between intestinal microbiota composition summarised to phylum level and cytokine levels revealed two significant correlations in the ileum. Levels of Firmicutes correlated negatively, and levels of Proteobacteria correlated positively with the expression of *il6* encoding IIL-6 measured in the mucosa (Table 1). The abundance of Verrucomicrobia correlated positively with expression of the gene *foxp3* coding for Foxp3 measured in the MLNs (Table 1). At the genus level, a majority of statistically significant correlations (19 out of 26) were found to be between specific taxa and the expression of genes related to anti-inflammatory or regulatory immunity (Table 1). Microbial composition in the ileum was correlated to gene expression in both the ileal mucosa and MLNs and resulted in 10 significant correlations between bacterial abundances and MLN gene expression. No correlation to gene expression in the ileal mucosa was found at the genus level. Cecal and colonic microbial compositions were correlated to gene expression in the MLN, and 14 significant correlations were observed for the cecal microbiota, while only two for the colonic. All correlations can be found in Table 1. Increasing abundances in both ileum and cecum of several Colstridiales spp. correlated positively with increased expression in MLN of the regulatory T-cell transcription factor *foxp3* gene or the *il10* gene encoding anti-inflammatory IL-10. An unclassified Ruminococcaceae correlated negatively with the expression of *il17*, a pro-inflammatory gene produced by T-helper cell type 17 (Th17). Positive correlations were found between increasing abundances of *CF213* (Bacteroidetes) and the anti-inflammatory gene *tgfb*. Finally, increased levels of an unclassified member of the Desulfovibrionaceae family (Proteobacteria) correlated positively with the expression of *il10*, *foxp3*, and *tgfb* in the MLN.

**Table 1:** The table shows significant correlations (p > 0.05), in a Speerman Rank Correation, at the Phylum and genus level.

Level	Compartment	Effect	Taxa				Cytokine	Tissue	Corellation coeff	p value
			Phylum	Order	Family	Genus				
	ileum	Anti-inflammatory	Firmicutes				*il6*	*mucosa*	−0.47218	0.0503
			Verrucomicrobia				*foxp3*	*mln*	0.43337	0.0497
		Pro-inflammatory	Proteobacteria				*il6*	*mucosa*	0.30376	0.0381
	ileum	Anti-inflammatory	Spirochaetes	Spirochaetales	Spirochaetaceae	*Treponema*	*foxp3*	*mln*	0.45015	0.0406
					Unclassified		*foxp3*	*mln*	0.51818	0.0161
					[Mogibacteriaceae]	Unclassified	*foxp3*	*mln*	0.44699	0.0422
					Peptostreptococcaceae	Other	*foxp3*	*mln*	0.55038	0.0097
						Ulclassified	*foxp3*	*mln*	0.55325	0.0093
			Firmicutes	Clostridiales	Lachnospiraceae	*Ruminococcus*	*foxp3*	*mln*	0.49593	0.0222
						*Roseburia*	*foxp3*	*mln*	0.51891	0.0159
						*Dorea*	*foxp3*	*mln*	0.56303	0.0079
					Ruminococcaceae	Unclassified	*foxp3*	*mln*	0.46004	0.0359
						*Oscillospira*	*foxp3*	*mln*	0.49887	0.0213
	cecum	Anti-inflammatory				*Dorea*	*il10*	*mln*	0.42192	0.0400
					Lachnospiraceae	*Coprococcus*	*il10*	*mln*	0.45749	0.0246
			Firmicutes	Clostridiales		*Blautia*	*il10*	*mln*	0.43183	0.0351
					Ruminococcaceae	Unclassified	*il17*	*mln*	−0.52647	0.0362
						*Oscillospira*	*foxp3*	*mln*	0.53014	0.0077
			Bacteroidetes	Bacteroidales	[Paraprevotellaceae]	*CF231*	*tgfb*	*mln*	0.53652	0.0069
							*il10*	*mln*	0.60945	0.0016
			Proteobacteria	Desulfovibrionale	Desulfovibrionaceae	Unclassified	*tgfb*	*mln*	0.56850	0.0037
							*foxp3*	*mln*	0.45568	0.0252
		Pro-inflammatory	Firmicutes	Clostridiales	Lachnospiraceae	*Coprococcus*	*il6*	*mln*	0.46227	0.0229
					Veillonellaceae	*Phascolatobacterium*	*il6*	*mln*	0.44100	0.0314
			Bacteroidetes	Bacteroidales	[Paraprevotellaceae]	*CF231*	*il6*	*mln*	0.44000	0.0314
							*il12*	*mln*	0.53652	0.0069
			Proteobacteria	Desulfovibrionale	Desulfovibrionaceae	Unclassified	*il12*	*mln*	0.56850	0.0037
	colon	Pro-inflammatory	Firmicutes	Clostridiales	Lachnospiraceae	*Coprococcus*	*il6*	*mln*	0.45532	0.0254
			Bacteroidetes	Bacteroidales	[Paraprevotellaceae]	*[Prevotella]*	*foxp3*	*mln*	−0.62647	0.0011

At the phylum level, significant correlations were found between the expression of genes encoding the pro-inflammatory cytokine IL-6 and regulatory T-cell transcription factor Foxp3 in the ileal mucosa and MLN and phylum abundances. At the genus level, taxa present in more than 50% of all samples and with an abundance above 0.1% in the gastrointestinal tract of horses were used for the analysis. Significant correlations were found between bacterial abundances in ileum, cecum, and colon, and the expression of genes encoding the two anti-inflammatory cytokines IL-10 and TGF-β, the four pro-inflammatory cytokines IL-6, IL-12, IL-17 and TNF-α, and regulatory T-cell transcription factor Foxp3.

### Firmicutes is the dominant phylum in the horse gastrointestinal tract although the microbiota significantly differs between the ileum, cecum, and colon

**Figure 1:**
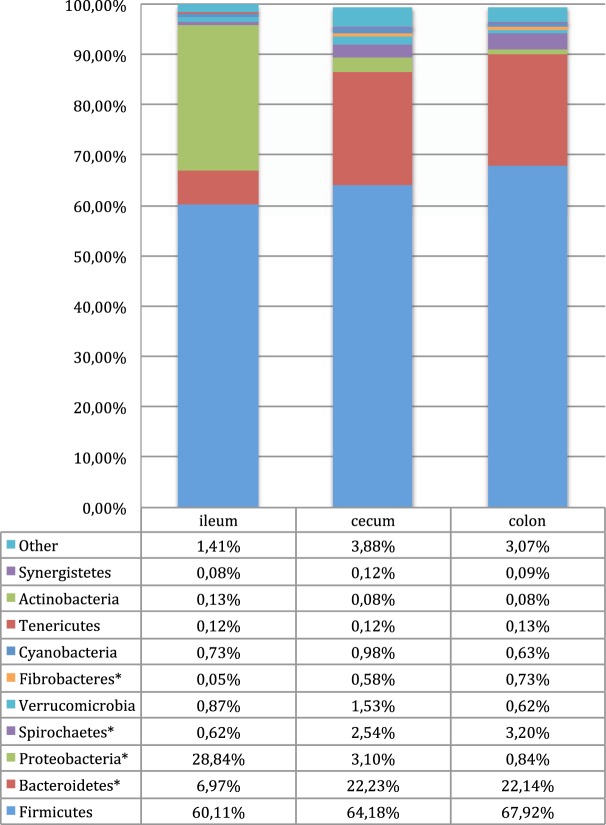
Phyla identified by amplicon sequencing of the V3-V4 region of the 16S rRNA gene and percentage abundance based on normalized OTU numbers in the ileum, cecum and colon of 24 horses. Phyla with abundance above 0.1% in at least one compartment are shown separately. The group “Other” includes the phyla Elusimicrobia, Fusobacteria, Planctomycetes, SR1, TM7, and WPS-2 all with abundances below 0.1%. Samples from 21 horses were used to analysis of the microbial composition in the Ileum and from 24 horses for the Cecum and Colon. Significant differences were found between ileum and the hind gut (cecum/colon), but not between the cecum and colon in an ANOVA analysis. Phyla that differed significantly are marked with *(p < 0.05).

**Figure 2:**
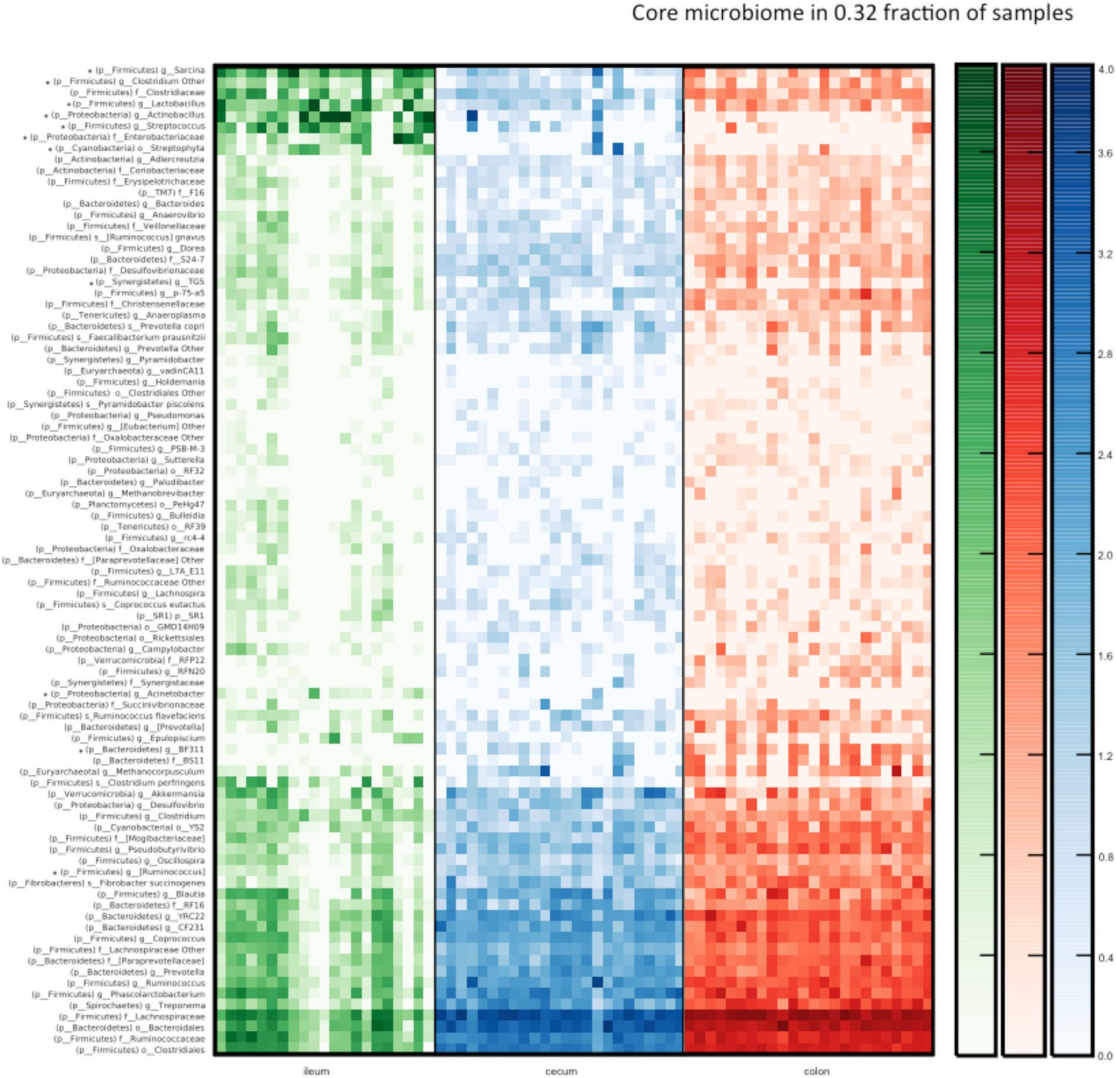
Heatmap illustrating a core microbiome (taxa represented in at least 32% of all samples) characterized with amplicon sequencing of 16S rRNA gene (V3-V4 region) within three sections of horse gastrointestinal tract. *Indicates taxa with significantly shifted abundance between the three sections (ANCOM, FDR p < 0.05). No major differences in the taxa abundance were detected between the colon and cecum microbiota composition. Scale on the right indicate log abundance.

Ten phyla were consistently detected across all sample sites (ileum, cecum, and colon) with a mean abundance above 0.1% (Fig. 1). These were Firmicutes, Bacteroidetes, Proteobacteria, Spirochaetes, Verrucomicrobia, Fibrobacteres (mean abundance ileum 0.05%), Cyanobacteria, Tenericutes, bacteria (mean abundance cecum and colon 0.08%) and Synergisteres (mean abundance ileum 0.08% and colon 0.09%). Additionally, Planctomycetes and candidate phyla SR1, TM7 and WSR-2 were detected in all compartments although at abundances below 0.1%. Firmicutes was the most abundant phylum in all three intestinal compartments followed by Proteobacteria in ileum and Bacteroidetes in the cecum and colon (Fig. 1). Proteobacteria were significantly (p = 0.0001) more abundant in the ileum compared to the cecum and colon, and Bacteroidetes were significantly more abundant in the cecum and colon compared to the ileum (p = 0.0001). Compared to the ileum, Fibrobacteres and Spirochaetes were significantly (p = 0.02 for Fibrobacteres, p = 0.01 for Spirochaetes) more abundant in the colon, and in both cecum and colon respectively (Fig. 1). A core equine microbiota was defined on the genus level. A core microbiota member was defined as a taxa present in at least 32% of the samples. This definition was decided upon before the data analysis. 32% was chosen to both ensure a general high abundance in the samples and at the same time ensure that taxa present in high abundance in only one of the three compartments would still be included in the core microbiota (Fig. 2). Significant differences in abundances of the core bacteria were present between the compartments, primarily between the ileum and cecum/colon. Within Firmicutes, ANCOM analysis (Fig. 2) demonstrated significantly (p < 0.05) higher abundances of *Lactobacillus*, *Streptococcus*, unclassified *Clostridium* and *Sarcina* in the ileum, and a significantly (p < 0.05) higher abundance of members of *Ruminococcus* in both cecum and colon, compared to the ileum. The higher abundance of Proteobacteria in the ileum was caused by significantly higher abundances (p < 0.05) of *Actinobacillus*, *Acinetobacter* and Enterobacteriaceae compared to the cecum and colon (Supplementary Fig. S2). In the cecum and colon, Bacteroidetes was the second most prominent phylum with the candidate genera BF311 being significantly (p < 0.05) more abundant than in the ileum. *Streptophyta* belonging to Cyanobacteria and TG5 belonging to Synergisteres differed significantly between the colon and ileum, but not between the ileum and cecum (Supplementary Fig. S2).

## Discussion

The aim of this study was to investigate the influence of the horse’s intestinal microbiota on the expression of genes signifying regulatory immunity. Our results show a significant correlation between certain bacterial species and the regulatory gene expression profile in the horse GIT, indicating that these species may induce regulatory immunity. We found a potential regulatory effect of Verrucomicrobia in the ileum on the up-regulation of *foxp3*. Verrucomicrobia and particularly the species *Akkermansia muciniphila (A*. *muciniphila)* have been shown to induce regulatory immunity in mice and to have a positive impact on diseases modulated by low-grade chronic inflammation^18,55,56^. *A*. *muciniphila* inhabits the mucous layer of the GIT and modulates pathways involved in immune regulation toward oral tolerance to commensal bacteria^57^. In the present study we found *A*. *muciniphila* in the ileum and a trend towards a positive correlation with *foxp3* in the MLN (p = 0.0561) this may indicate that other members of the Verrucomicrobia phylum also contribute to the positive correlation with *foxp3*. *Foxp3* is involved in differentiation of regulatory T-cells mediating tolerance toward commensals, and *A*. *muciniphila* has been shown to induce differentiation of Foxp3 positive regulatory T-cells^18^. The present study suggests that *A*. *muciniphila* also have beneficial immune properties in horses. However, further studies should be conducted to confirm the mucin degrading and immune modulating properties of *A*. *muciniphila* in horses on the molecular level. Overall we found Firmicutes to correlate with decreasing levels of the gene *il6* encoding the pro-inflammatory cytokine IL-6. A closer look at members of the Firmicutes phylum showed that Clostridiales spp. might play a prominent role in inducing regulatory immunity. Increasing abundance of eleven Clostridiales spp. (an unclassified member of Clostridiales, Rumminococcaceae, Lachnospiraceae, Mogibacteriaceae, and Peptostreptococcaceae, and *Ruminococcus*, *Roseburia*, *Dorea*, *Oscillospira*, *Coprococcus*, and *Blautia*) correlated positively with expression of the gene encoding regulatory T-cells transcription factor *foxp3* or the gene encoding the anti-inflammatory cytokine IL-10 or both (Table 1). In addition, increasing levels of unclassified Ruminococcaceae (also of the Clostridiales order) correlated negatively with expression of the pro-inflammatory cytokine *il17* gene (Table 1) supporting our theory that members of this order may indeed have regulatory properties.

In humans, a low abundance of Clostridiales spp. has been linked to pathological conditions, such as irritable bowel syndrome^58^. Especially the Clostridiales member *Faecalibacterium prausnitzii* has been found to be less abundant in human patients with Chrohn’s disease^59^ or obesity^60^. Concurrently reduced abundance of both Lachnospiraceae and *Ruminococcus* (both Clostridiales) have been found in horses with Equine Metabolic Syndrome (EMS)^61^. As in humans, EMS is a multifactorial disease characterized by a low-grade chronic inflammatory state, obesity, and insulin resistance^62,63^. An increase in the relative abundances of bacterial taxa associated with regulatory responses such as bifidobacteria has preventive and curative effects on the low-grade inflammation in human metabolic syndrome^19^. The decrease of Clostridiales members in the GIT of horses suffering from EMS, and the positive correlations between abundances of Clostridiales and markers of regulatory immunity, imply that members of this order have similar beneficial effects in horses as *Bifidobacterium* have in humans. Not all members of the Clostridiales order may be equally good at inducing regulatory immunity. *Coprococcus* abundances correlated with both anti-inflammatory *il10* and *il6* encoding interleukin IL-6 which can induce Th17 differentiation and is involved in upholding the Treg/Th17 balance^64^. In addition increasing abundances of *CF231* correlated with expression of *tgfb* known inducer of Treg, but also with the pro-inflammatory genes *il6* and *il12* in the MLN. This underlines the multifaceted relationship between intestinal microbiota and the immune system and the sensitivity of the immunological homeostasis to the surrounding cytokine, chemokine, and microbial stimuli.

As in previous studies attempting at establishing a common microbiome of other mammalian species we found Firmicutes to be the most abundant phylum in all three compartments^30–35^, but with a significant difference between the ileum and the hindgut (cecum, colon) (Fig. 1). The majority of the differences were found as significant differences in abundances of the phyla Firmicutes, Bacteroidetes, and Proteobacteria. This apparent difference between the ileum and the hindgut seems logical as there are important physiological and digestive differences between the two parts of the intestinal tract, with the hindgut being the main place of bacterial fermentation. In the ileum Proteobacteria was the second most abundant phylum representing 28.8% of the taxa. High abundances of Proteobacteria have been associated with intestinal inflammatory diseases in mice, humans, and cattle^15,65,66^. However high abundances of Proteobacteria in the upper part of the gastrointestinal tract of clinically healthy horses as observed in this study, have also been observed by others^30,37^. On the phylum level we saw a positive correlation between Proteobacteria levels and *il6* expression, but on the genus level no correlations were found between Proteobacteria in the ileum and the expression of the cytokine-encoding genes. In the cecum and colon Proteobacteria were much less abundant, and one member of the phylum and unclassified Desulfovibrionaceae positively correlated with increased expression of both pro-inflammatory *(il6)* and anti-inflammatory *(il10*, *tgfb*, *foxp3)* cytokines. The presence of Proteobacteria thus seems to have some immunological effects, but the high abundance found in the ileum in healthy horses in this study, and others lead us to believe that they are part of the normal microbiota. Bacteroidetes was the second largest phylum in the hindgut and significantly more abundant in the hindgut compared to the ileum. At the genus level the Bacteroidetes spp. *BF311* was significantly increased in the colon compared to the ileum. Two previous studies have found significant increases in *BF311* abundance when horses were turned to a more fibre-rich diet^8,67^. This suggests that members of the Bacteroidetes phylum are important for the digestion of fibres and for maintaining a healthy intestinal microbiota in the hindgut. While *BF311* correlated positively with anti-inflammatory *tgfb*, it also correlated positively to pro-inflammatory cytokines *il6* and *il12*, and therefore this was not considered an ideal candidate for increasing regulatory immunity.

The above findings suggest that Verrucomicrobia and Clostridiales spp. are potential targets for inducing regulatory immunity. The only described Verrucimicrobia member having the GIT as its ecological niche is *A*. *muciniphila*, which also have been shown to be promoted by oligosaccharide feeding^68^. Clostridiales play an important role in the digestion of poly- and oligosaccharides^69^, and *Ruminococcus* (belonging to the Clostridiales) has previously been identified as major fibre fermenters in the equine GIT^32,70,71^. Oligosaccharides may therefore, be a possible stimulator of Verrucomicrobia spp. and Clostridiales spp. growth in the equine GIT and subsequently have a positive impact on the immune-microbial homeostasis.

As the aim was to describe a core microbiome and to study immune-microbial interaction in the general equine population, the varied nature of the horses and uncontrolled diet, age, and sex, may very well be a good representation of a typical equine population. However the study has several limitations and additional studies should be conducted to confirm the results and to address the limitations mentioned below. It would have been beneficial if the information on certain factors such as diet had been available because the diet has been shown to affect the microbiota^25,67^, as has age, as the not fully established microbiota in younger horses differ from that of older horses^72^. Studies suggest that the equine gut microbiota stabilises within the first year of life^72–74^. The youngest horse in this study was two years of age and we therefore expect all horses to have an adult-like microbiota. Additionally age and gender may have an effect on the microbiota composition, and further studies should be conducted to clarify this. To our knowledge this is the first study investigating the immune microbial interactions in the equine gut. Further studies should be conducted to investigate the regulatory and anti-inflammatory potential of the identified bacteria, preferably by using a broader spectrum of immune markers. Based on the knowledge from this study *in vitro* fermentation experiments and studies investigating the immune cell distribution locally and systemically would help to further elucidate the immunological impact of the microbiota.

## Conclusion

In conclusion, we showed that members of the intestinal microbiota, especially Verrucomicrobia spp. and Clostridiales spp., are associated with increased expression levels of the regulatory cytokines *il10* and *tgfb* and the Treg transcription factor *foxp3* in the horse. These bacteria may therefore, be possible candidates for induction of regulatory immunity in the host. We identified a core microbiota in the equine GIT with significant differences in composition between the ileum and the hindgut. These results suggest that the species of the intestinal microbiota identified above have the potential to be useful targets for increasing of the regulatory immunity in the GIT.

## Supplementary information

Supplementary information

## Data Availability

Data and associated protocols are stored on the University of Copenhagen’s backup servers, the sequencing data is available in the European Nucleotide Archive (EAN) with the study number PRJEB33830 and the Q-PCR data at Figshare, 10.6084/m9.figshare.9311813.
