# Supplementary material for: Expression of immune regulatory genes correlate with the abundance of specific Clostridiales and Verrucomicrobia species in the equine ileum and cecum
Source: Sci Rep. 2019 Sep 3;9:12674. doi: 10.1038/s41598-019-49081-5 (PMC6722064; doi:10.1038/s41598-019-49081-5)
Supplement: Supplementary file 1 — Supplementary information [file 41598_2019_49081_MOESM1_ESM.pdf]

Expression of immune regulatory genes correlates with the abundance of specific Clostridiales and Verrucomicrobia species in the equine ileum and cecum

F. Lindenberg[1,2*], L. Krych[4], J. Fielden[1], W. Kot[5], H. Frøkiær[2], G. van Galen[3], D. S. Nielsen[4], A. K. Hansen[2]

[1] Brogaarden Aps

[2] University of Copenhagen, Faculty of Health and Medical Sciences, Department of Veterinary and Animal Sciences

[3] University of Copenhagen, Faculty of Health and Medical Sciences, Department of Veterinary Clinical Sciences

[4] University of Copenhagen, Faculty of Sciences, Department of Food Science

[5] Department of Environmental Sciences, Aarhus University

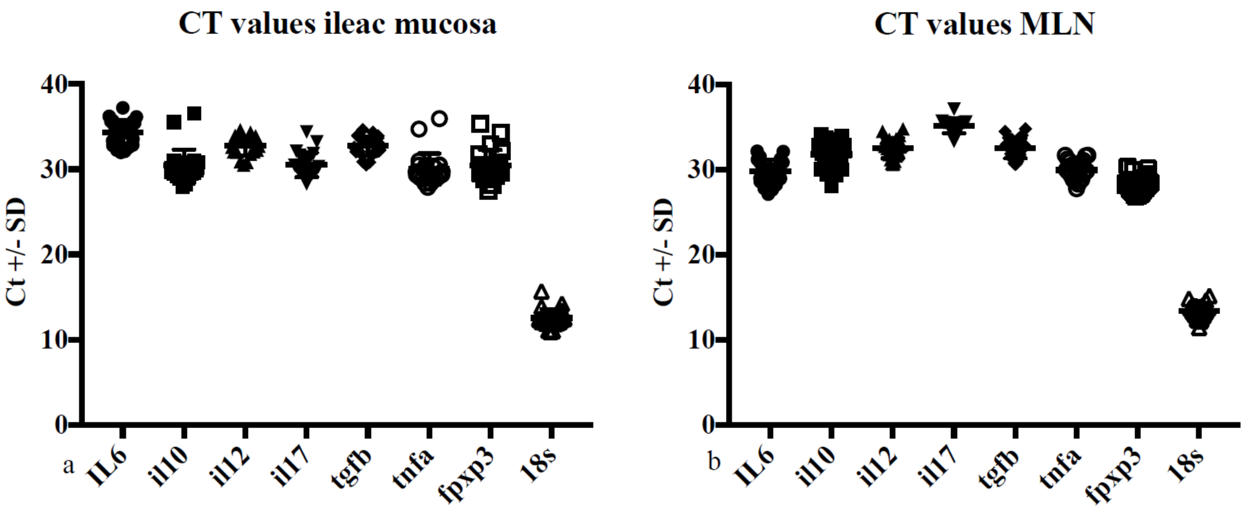

Supplementary figure S1: Gene-expression of *foxp3, tnfa, tgfb, il17, il12, il10*, and *il6* in ileal mucosa (a) and mesenteric lymph nodes (b) measured by qPCR and expressed as mean Ct +/- SD. In horse number 1 and 2 ileal expression of *il12, il17,* and *tgfb* and *il6, il1,2* and *il17* respectively were not detected for unexplained reasons nor was expression of *il17* in horse 24. In the colon *il17* expression was not detected in horses number 4, 5, 11, 17, 20, 22, 23, and 24.

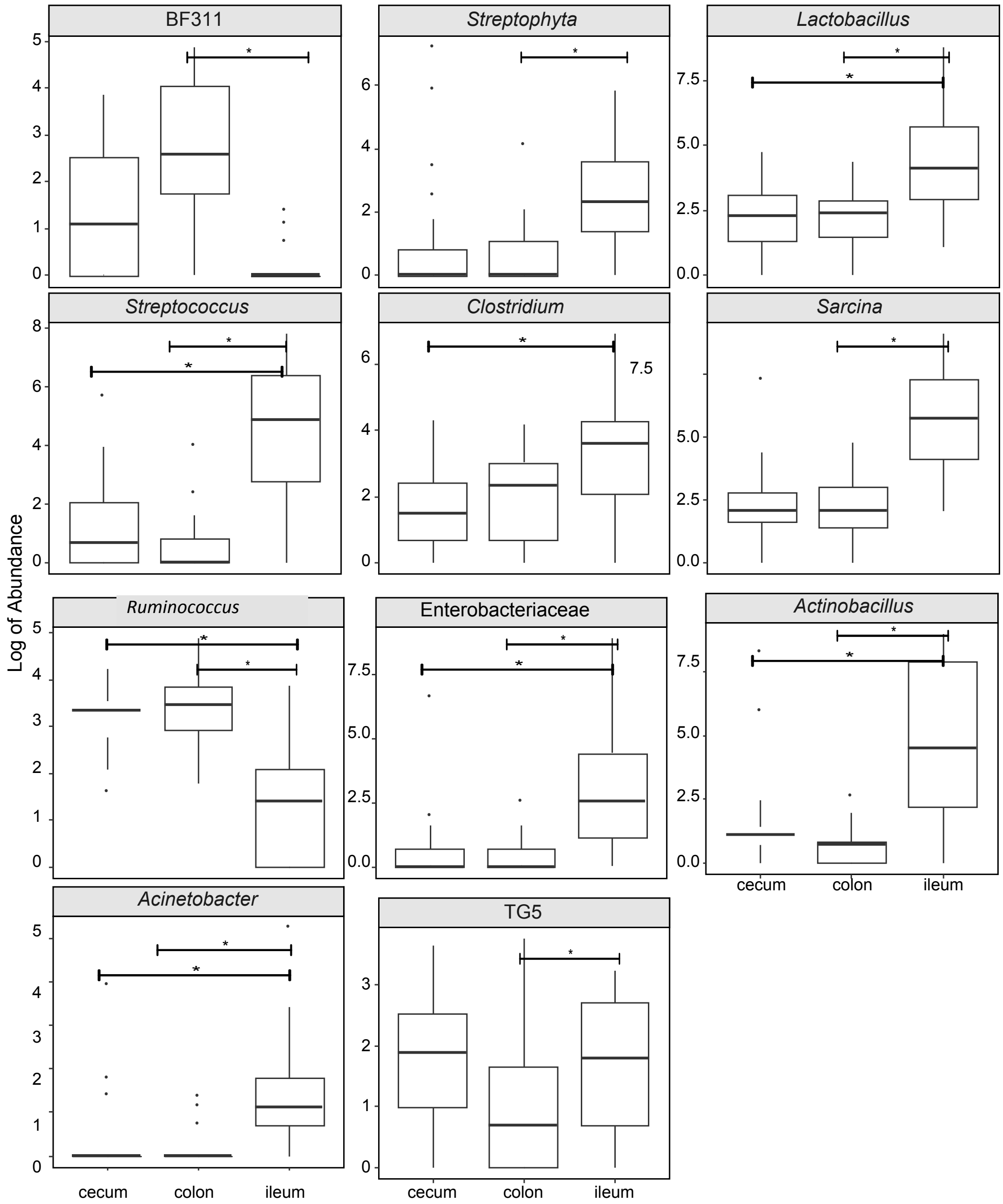

Supplementary Figure S2:

Figures shows box plots of species found to differ significantly between the ileum, cecum, and colon. Significantly different abundances were found between the ileum and the cecum and the ileum and colon, denoted by * (ANCOM, FDR $p < 0.05$). There were no major differences observed between the colon and the cecum microbiota composition. Box plots show log abundance (ANCOM analysis) on amplicon sequencing of 16S rRNA gene (V3-V4 region) within three sections of horse gastrointestinal tract (box limits represent upper and lower quartiles, bold line the median, whiskers the 1.5 IQR and dots the extremes).
